# Probiotics maintain the gut microbiome homeostasis during Indian Antarctic expedition by ship

**DOI:** 10.1038/s41598-021-97890-4

**Published:** 2021-09-22

**Authors:** Ashish Kumar Srivastava, Vishwajeet Rohil, Brij Bhushan, Malleswara Rao Eslavath, Harshita Gupta, Sudipta Chanda, Bhuvnesh Kumar, Rajeev Varshney, Lilly Ganju

**Affiliations:** 1 Defence Institute of Physiology and Allied Sciences (DIPAS), Defence Research and Development Organization (DRDO), Lucknow Road, Timarpur, New Delhi 110054 India; 2 Department of Biochemistry, Vallabhbhai Patel Chest Institute, University of Delhi, Delhi, 110007 India

**Keywords:** Microbiology, Sequencing

## Abstract

Ship voyage to Antarctica is a stressful journey for expedition members. The response of human gut microbiota to ship voyage and a feasible approach to maintain gut health, is still unexplored. The present findings describe a 24-day long longitudinal study involving 19 members from 38th Indian Antarctic Expedition, to investigate the impact of ship voyage and effect of probiotic intervention on gut microbiota. Fecal samples collected on day 0 as baseline and at the end of ship voyage (day 24), were analyzed using whole genome shotgun sequencing. Probiotic intervention reduced the sea sickness by 10% compared to 44% in placebo group. The gut microbiome in placebo group members on day 0 and day 24, indicated significant alteration compared to a marginal change in the microbial composition in probiotic group. Functional analysis revealed significant alterations in carbohydrate and amino acid metabolism. Carbohydrate-active enzymes analysis represented functional genes involved in glycoside hydrolases, glycosyltransferases and carbohydrate binding modules, for maintaining gut microbiome homeostasis. Suggesting thereby the possible mechanism of probiotic in stabilizing and restoring gut microflora during stressful ship journey. The present study is first of its kind, providing a feasible approach for protecting gut health during Antarctic expedition involving ship voyage.

## Introduction

Antarctic ship voyage can increase risks to personal health and wellbeing. However, it maintains the supply of essential goods and other necessities to Antarctic expedition members who work at Antarctic stations and on-board. The Antarctic and Southern Ocean environment includes low temperature, blizzards, intense UV radiations, high humidity and salinity, isolation, sleep deprivation, altered circadian rhythms, sea sickness and unavailability of fresh fruits and vegetables which leads to psychological and physiological stress^1,2^. Expedition members under these conditions are exposed to health complications as compared to mainland workers^3^. Also, the challenging conditions encountered during the ship voyage to Antarctica greatly affect human performance due to the presence of various environmental and social factors such as isolation, fear and confinement^4,5^. Environmental factors could lead to alterations of the immune^6,7^ and digestive system. In addition, septicemia caused by vitamin deficiency is the greatest danger to sailor’s health^8^. Sea-sickness, loss of appetite, nausea, and fear of unknown are also possible threats for the development of adverse health problems  that could be related to altered gut microbiome. The role of the human microbiome in human health is essential for the regulation of immune system^9,10^, nutrient absorption^11^, maintaining homeostasis^12^, functions of vital organs, including the liver^13–15^, kidney^16^ and the brain^17–20^ etc. Since, most of the expedition members in 38th expedition were travelling for first time by ship in the extreme stressful environment of Antarctica, various health issues were prominent in most of the members.

Evidences from recent studies suggest the importance of diversity in gut microbial species and functional genes in various chronic metabolic diseases^21^. However, studies on health management during Antarctic expedition by ship voyage remains to be explored in detail. Limited studies available regarding the ship voyage and stay at Antarctica have reported alterations in both gut^22^ and salivary microbiome^4^ but its fixation/restoration via any means has not been explored yet. Probiotic intervention could be the most suitable, convenient and appropriate approach which could help maintain the balance of micro-ecology and prevent secondary infections. Probiotics alter the composition of gut microbiota by increasing beneficial microbes and decreasing pathogen colonization^23,24^. Functional effects of probiotic strains on gut microbiota composition and related diseases have been clearly illustrated however, some studies have shown that intestinal microbiota have a less prominent response to oral probiotics, possibly due to inter-individual variation in the composition of microbiota produced by diet, genetics, antibiotic use, health history, age, and other environmental factors. Although not all of these findings were optimistic, Zhang et al.^25^ reported that probiotics exhibit common effects on the regulation of gut microbiota during ship voyage. In the present study, the primary goal was to investigate—if probiotics could modulate intestinal microbes towards a more favorable balance and strengthen immunity during ship voyage to Antarctica under extreme environmental conditions.

## Materials and methods

### Subjects and experimental design

Nineteen Indian healthy members who participated in 38^th^ Indian Scientific Expedition to Antarctica via ship voyage volunteered (age, 27–45 years; all males) for the study. The participants were enrolled for the study after obtaining written informed consent. Subsequently, the volunteers were randomly divided into two groups: the placebo (PCB) group (n = 09) and probiotic (PB) group (n = 10). Probiotic intervention was continued for 24 days (from January to February), which allowed sufficient duration for intestinal flora to change. Probiotic consumption was strictly monitored on a daily basis during the ship voyage till the completion of the voyage. Being a vessel ship on expedition, there was not enough choice of food items available, nor was it possible to carry variety of items because of logistics problems. The food items were available as buffet with different menu every day, though repeated on a weekly basis. Everyone was served the similar food available on board. The difference between vegetarian and non-vegetarian food items was minimum since the majority of items were common. The demographic data of 19 study participants is shown in Table 1, individual expedition member data across all the time points and details of vegetarian, non-vegetarian and combined food items available on-board during the ship voyage is depicted in Supplementary Tables S1 and S2, respectively.

**Table 1:** Demographic data for 19 study participants.

Parameters	Values (n = 19)	
	Placebo group (n = 09)	Probiotic group (n = 10)
Age-yrs, median (range)	31 (27–40)	32.50 (27–42)
Weight-kg, median (range)	75 (68–95)	76.50 (60.6–114)
BMI-kg/m^2^, median (range)	26.10 (22.70–31)	26.15 (21.10–33)
SBP-mmHg, median (range)	123 (112–146)	123 (111–132)
DBP-mmHg, median (range)	78 (70–86)	73 (62–87)
Pulse rate-per min, median (range)	77 (53–94)	74.5 (47–84)
**Diet pattern %**		
Vegetarian	22.2	20
Non-vegetarian	77.7	80
Sea sickness %	44.4	10

### Ethical statement

The experimental study was approved by the Research Ethics Committee of Defence Institute of Physiology and Allied Sciences, Defence Research and Development Organization, New Delhi, India. All participants understood the nature of study and provided informed written consent. The study was conducted in accordance with the ethical standards of 1964 guidelines of the Declaration of Helsinki.

### Probiotic composition

The probiotic used was a commercially available lyophilized powder (LifeZen, WONDERPRO, 1 gm sachet, composed of sorbitol, xylitol, maltodextrin, stabilizers, anti-caking agents, in addition to 5 × 10^9^ CFU/g bacteria), enriched with *Lactobacillus acidophilus* (1.6 × 10^9^ CFU/g), *Lactobacillus rhamnosus* (0.8 × 10^9^ CFU/g), *Bifidobacterium longum* (0.8 × 10^9^ CFU/g), *Saccharomyces boulardii* (0.2 × 10^9^ CFU/g), *Bacillus coagulans* (1.6 × 10^9^ CFU/g). PB group subjects were provided with one-gram of probiotic in sachet, daily morning, whereas PCB group was provided with one-gram of placebo in sachet consisting of sorbitol, xylitol, maltodextrin, stabilizers, anti-caking agents without any microbial strain.

### Sample collection

An early morning freshly voided stool sample was collected from each volunteer from both the groups at day 0 in NCPOR, Goa, as baseline sample (T1). From day 1, PB group subjects were provided with one-gram of probiotic daily morning and PCB group were provided with one-gram of placebo. The expedition members flew to Cape Town, South Africa from Goa, India, and boarded the ship for further journey to Antarctica. Ship reached Indian-Antarctic station, Bharati on day 24 and stool samples were collected on day 25 (T2) from both the groups. After collection, all the samples were mixed with RNA Later and stored at − 40 °C till further analysis.

### DNA extraction, library construction and sequencing

Microbial DNA was extracted from the fecal samples, using the Power Fecal DNA Isolation Kit (Mobio, USA), according to the manufacturer’s protocol. DNA library construction was performed following the manufacturer’s mentioned protocol. All the samples for whole genome shotgun sequencing libraries were constructed using Illumina Trueseq DNA library preparation kit. In brief, 0.5 μg of purified metagenomic DNA was used for library construction. Paired-end shotgun libraries with an insert size of ~ 300 bp for each sample  were constructed according to a standard True Seq WGS protocol provided by Illumina, Inc. (San Diego, CA, USA) and sequenced. Libraries were indexed using TruSeq DNA Single Indexes kit set A. Indexed libraries were pooled and sequenced in a single HiSEQ 2500 lane with a read length of 2 × 100 bases. Library quality for fragment size was checked on Bioanalyzer 2100 using high sensitivity DNA kit (Agilent technologies, USA). A Qubit Fluorometer (Invitrogen, USA) and a Stratagene Mx3000P Real-time PCR Cycler (Agilent, USA), were used for library quantification prior to cluster generation in a c-Bot automated sequencing system (Illumina, Inc.). There was a negative extraction control setup during extraction (Autoclaved Nuclease-Free Water; Thermofisher).

### Raw data processing

MOCAT2 v 0.2^26^ pipeline was used to process the obtained raw metagenomic data for removal of low-quality reads, adapter removal, decontamination by human DNA. Sequencing of fecal matter for gut microbiota generated approximately a total of 107 GB data for 38 fecal samples (approximately 2.8 GB data/sample) (Supplementary Table S3). QC stats of the data have been depicted in Supplementary Table S4.

### Sequence data processing

Taxonomic profiling was performed from the raw metagenomic sequencing reads by using the MOCAT2 pipeline^26^, using various applications for different steps. Quality filtering and trimming of the raw reads was done using FastX toolkit 0.0.13, followed by screening reads to a custom database (Hg19 genome) by SOAP aligner v2.21 to remove human DNA contamination. Obtained high quality reads were assembled into contigs and scaftigs by SOAP denovo v2.04 and gene prediction from the contigs was done using Metagenmark 1.0. After gene prediction, single copy marker gene was extracted using fetchMG v1.0, and this reference catalog was then clustered using CD-HIT v4.7. Constructed gene catalogs were annotated to align genes to orthologues groups of database eggNOG using DIAMOND v0.8.22. Filtered reads were mapped to the above reference catalog for estimation of functional abundance using SOAPaligner v2.21. Same filtered reads were mapped to a refMG catalog and mOTU catalog for estimation of taxonomic and mOTU abundance (Supplementary Table S10) using SOAP aligner v2.21 respectively.

### Microbial diversity and taxonomic analysis

Cumulative sum scaling (CSS) was followed by Total Sum Standardization (TSS) to remove biases introduced by TSS and log2 transformation to account for the non-normal distribution of taxonomic counts data. Taxonomic abundance data was then used in calypso web server-based tool^27^ for alpha diversity analysis across all time points and was calculated using Shannon index and Simpson’s index based on genus and species profiles. Differentially abundant & significant taxa were evaluated using the Wilcoxon rank sum test at a *p*-value cut off < 0.05 which was considered to be statistically significant for reproducibility. Beta diversity was calculated by weighted UniFrac distances based principal coordinate analysis (PCoA).

### Functional profiling and metabolic pathway analysis

High-quality sequencing data and sample information was used to perform advanced statistical analysis using FMAP (Functional Mapping and Analysis Pipeline) to identify differentially abundant Kyoto Encyclopedia of Genes and Genomes (KEGG) orthologies (KOs) and pathways (www.kegg.jp/kegg/kegg1.html). DIAMOND (version 0.7.10)^28^, (https://github.com/bbuchfink/diamond/tree/v0.7.10) was used to align high-quality sequencing data against KFU (KEGG Filtered UniProt) reference cluster of FMAP. The KOs abundance was calculated using FMAP with the KFU best-hit (e-value < 1e–3, percent identity > 80%) results and differential abundance was determined using Kruskal–Wallis rank-sum test. The format of differentially abundant KOs from FMAP was converted to STAMP (Statistical Analysis of Metagenomic Profiles)^29^ compatible format and used to investigate functional variations between two groups. Unclassified reads were retained and two-sided Welch’s t-test was used. CAZymes (carbohydrate-active enzymes)^30^ were predicted from amino acid sequences using the Hmmscan software in the HMMER 3.0 package^31^ to produce alignments with family-specific CAZymes HMMs in the dbCAN database. Alignments were filtered at E-value < 1e–5 and coverage > 0.30. CAZymes gene abundance were calculated by mapping high-quality sequencing data to CAZymes genes and alpha diversity were determined using ANOVA test. CAZymes genes heatmap was constructed using pheatmap R package. Reporter Z-scores were calculated to reveal alterations in enriched metabolic pathways between the placebo and probiotic groups. A reporter score of > 2.3 (90 percent confidence according to normal distribution) was therefore used as a threshold to distinguish between pathways significantly.

### Correlation and regression analysis of microbial taxa

We explored the correlation between microbial groups and sea sickness. The association between the microbial groups and sea-sickness was calculated by Spearman’s correlation coefficient using Calypso software^27^, and *p* < 0.05 was considered significant. A Univariate Mixed Effect Regression analysis was performed using Calypso software to take into account the longitudinal aspect for analyzing the relationship between the gut microbiota composition and other phenotypic data (such as diet, nausea, sea sickness, vomiting, headache). The mixed-effect regression analysis was performed using calypso to perform a pairwise comparison between the probiotic and placebo group microbiome profiles. *p*-value < 0.05 was considered to be statistically significant. Relative abundance after css + log transformation was used for the analysis and compared by mixed-effect linear regression, including groups as biological condition and sea sickness, headache, nausea, vomiting, diet as random effects in individual analysis. The expected *p*-value for the correlation between species and sea sickness was calculated, and multiple testing was corrected for using Bonferroni with false discovery rate (FDR) correction of the *p*-value at a 5% threshold. Phenotypic data was used in the form of yes or no.

### Statistical analysis

Differential abundance of phylum, genus, and species was tested by Wilcoxon rank-sum test between time points (PCB_T1, PCB_T2, PB_T1, and PB_T2) and PCB and PB using R script (version 3.5.2)^32^. Statistical analysis like principal component analysis (PCA) and principal coordinate analysis (PCoA) were executed using the Calypso software^27^. UniFrac was selected as a distance matrix for statistical analysis in Calypso software. Histogram bar plots were generated using Excel (MS Office 2010) and ggplot2 (R-package). Statistical Analysis of Metagenomic Profiles software (STAMP, version 2.1.3) was used for the pathway statistical analysis. Two-sided Welch’s t-test and Wilcox rank-sum test were applied for taxonomic differential abundance analysis between the two groups and within the groups, respectively. A two-sided Welch’s t-test was applied for differentially abundant pathways analysis. A *p*-value was also computed, indicating if the taxas were significantly associated with community composition. For all the analyses, *p* < 0.05 was considered to be statistically significant. The expected *p*-value for the correlation between species and sea sickness was calculated, and multiple testing was corrected for using Bonferroni with FDR correction of the *p*-value at a 5% threshold.

## Results

### Alteration of the gut microbiome in expedition members during ship voyage

**Figure 1:**
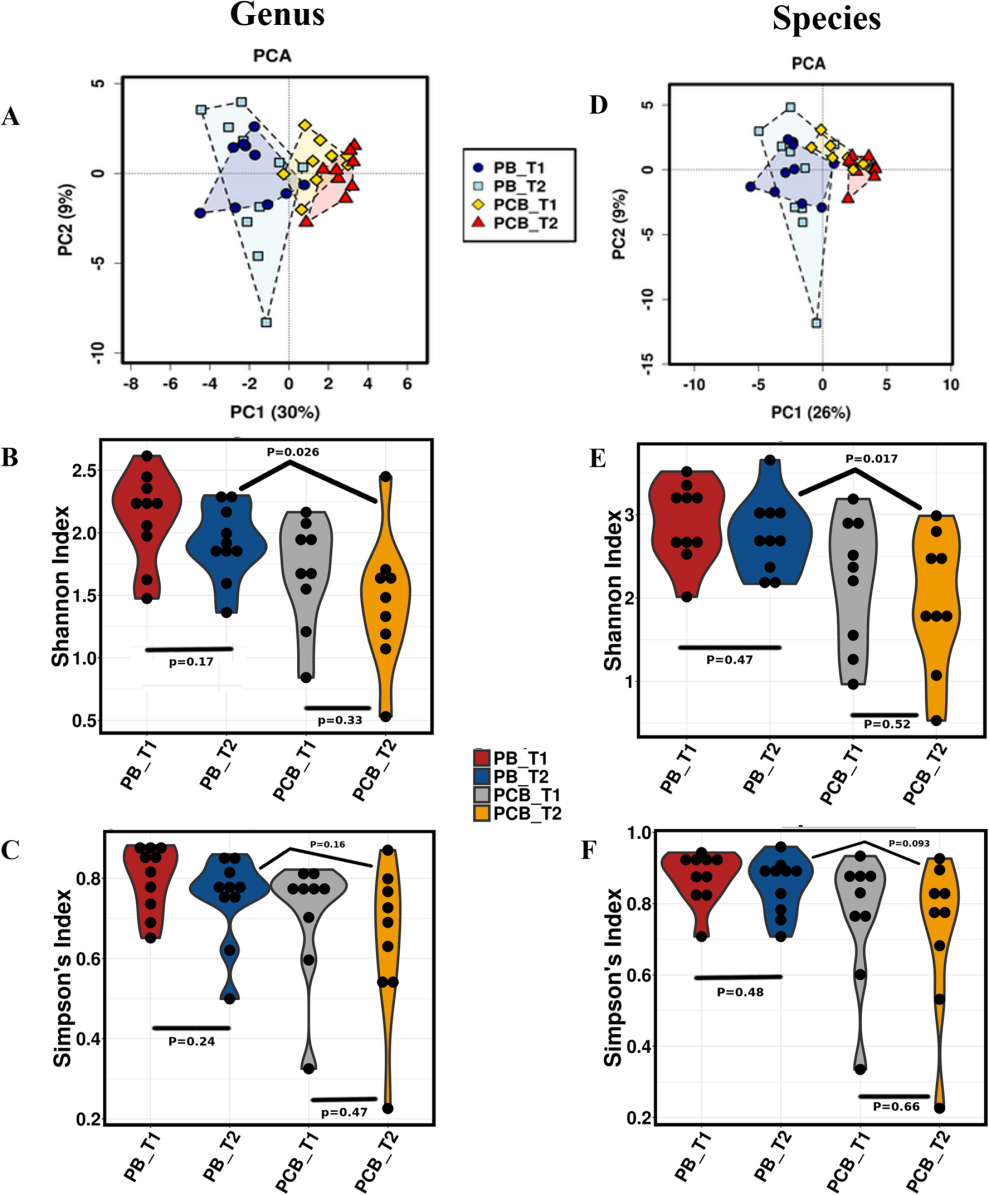
The impacts of a stressful ship voyage on the gut microbiota of expedition members. **A**, **D** - PCA of the gut microbiome in the  PCB and PB group between baseline (day 0) and after the completion of ship voyage (day 24) at genus and species level. **B**, **E** - Shannon index represented microbial alpha diversity of gut microbiome, within the groups and between the groups after the completion of ship voyage. Significant *p*-value (*p* < 0.05) was observed between PCB_T2 and PB_T2 at both genus and species level. ** C**, **F** - Simpson’s index represented microbial alpha diversity of gut microbiome, within the groups and between the groups after the completion of ship voyage. *p*-value < 0.05 was considered statistically significant.

A total of 428,852,502 (× 2 paired-end reads) reads for the fecal microbiota were analyzed with a mean of 2,751,879; 2,359,378; 22,596,826 and 15,688,293 reads for the PCB_T1, PCB_T2, PB_T1 and PB_T2 groups, respectively (Supplementary Table S3). Css + log normalized relative abundance was calculated at the genus and species level (Supplementary Table S8, S9). Based on the Bray–Curtis distances of the whole genome shotgun sequencing profiles at genus and species level, a PCA was performed (Fig. 1A, D). In terms of organism composition, the intestinal microbiota of subjects in the  PCB group at day 0 and day 24 was distinct, which indicated the impact of stressful ship voyage on the gut microbiota of expedition members. The α-diversity were reported as Shannon coefficients and Simpson’s index. A significant change was observed between PCB_T2 and PB_T2 groups after the completion of the journey, according to Shannon index at genus and species level (p = 0.026 and 0.017 respectively) whereas, no significant change was observed within the groups. On the other hand, no significant difference was observed according to Simpson’s index (Fig. 1C, F). In Shannon index we observed a sharp decrease in microbial alpha diversity between the members at baseline and at the end of ship voyage (Fig. 1B, E, Supplementary Table S5).

**Figure 2:**
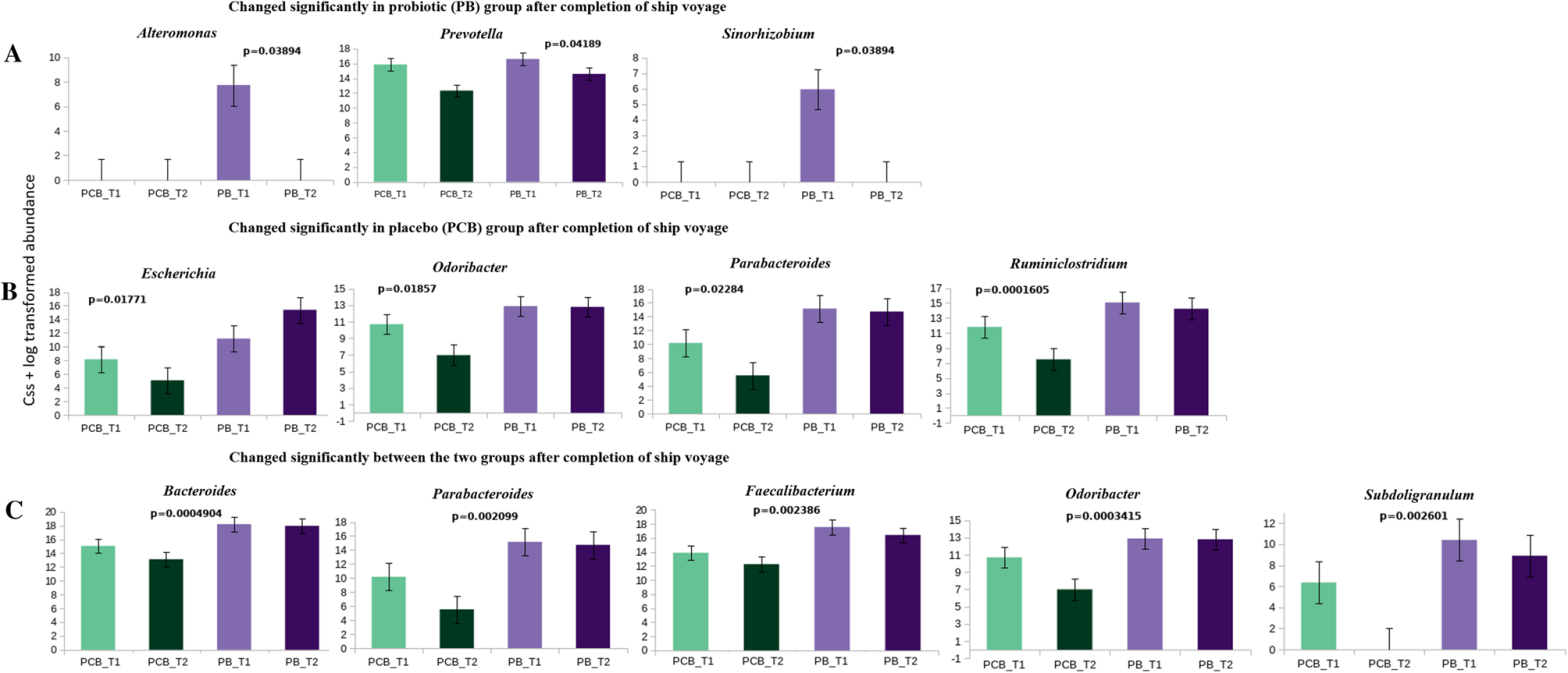
Taxonomic composition (css + log transformed) at genus level. **A** - At baseline, the genus did not have a significant difference between PCB and PB groups, but changed significantly in the PB group at the end of ship voyage. **B** - The genus changed significantly only in PCB group during the ship voyage. **C** - The genus abundance changed significantly between PCB and PB groups after completion of the ship voyage (PCB_T2 and PB_T2). *p*-value < 0.05 was considered to be statistically significant (Wilcoxon rank-sum tests) using in-house R-script. Error bar represent standard error mean (SEM).

**Figure 3:**
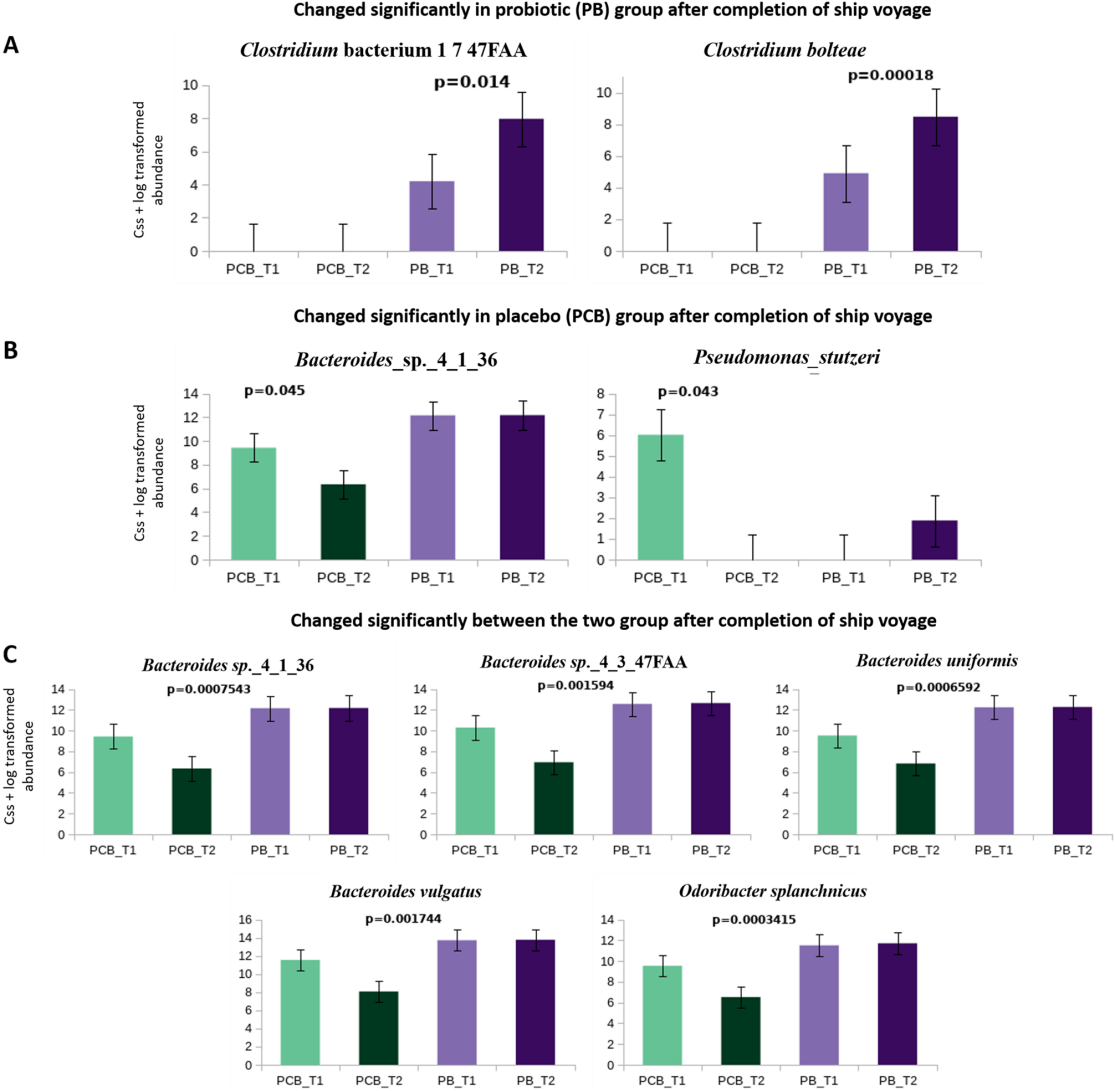
Taxonomic composition (css + log transformed) at species level. **A** - At baseline, the species did not have a significant difference between PCB and PB groups, but changed significantly in PB group at the end of ship voyage. **B** - The species changed significantly only in PCB group during the ship voyage. **C** - The species abundance changed significantly between PCB and PB groups after completion of the ship voyage (PCB_T2 and PB_T2). *p*-value < 0.05 was considered to be statistically significant (Wilcoxon rank-sum tests) using in-house R-script. Error bar represents standard error mean (SEM).

At the taxonomic level (Fig. 2, Supplementary Tables S8 and S9), we focused on the intestinal genus and species that had no significant differences between the PCB and PB groups at baseline but changed significantly at the end of ship voyage T2 (day 24) in the PCB and PB group. To correct the potential bias introduced by the relative microbial abundance, we performed a cumulative sum scaling (css) + log transformation of the relative abundance matrix. By comparing the genus abundance at baseline, we observed that the css + log abundances of genus *Escherichia, Odoribacter, Parabacteroides* and *Ruminiclostridium*, decreased significantly in expedition members of the PCB group at the end of ship voyage (Fig. 2B), whereas the css + log abundances of the species *Bacteroides sp*. 4_1_36 and *Pseudomonas stutzeri* were significantly declined (Fig. 3B; Wilcoxon rank-sum tests). Meanwhile, we compared the differences in genus and species abundance between PCB and PB groups at the end of ship voyage T2 and found that the genus *Bacteroides, Parabacteroides, Faecalibacterium, Odoribacter* and *Subdoligranulum* increased sharply in PB group (Fig. 2C). Whereas species *Bacteroides sp*. 4_1_36, *Bacteroides sp*. 1_3_47FAA, *Bacteroides uniformis*, *Bacteroides vulgatus* and *Odoribacter splanchnicus* were increased significantly in PB group (Fig. 3C; Wilcoxon rank-sum tests).

### Probiotics maintained gut microbiome homeostasis in expedition members during the ship voyage

Relative abundance of PCB and PB group was calculated at phylum, genus and species level (Relative abundance phylum and genus Supplementary Tables S6, S7, Supplementary Fig. SF1; css + log normalized abundance genus and species Supplementary Tables S8 and S9). Since we observed that the stressful ship voyage had a significant impact on the gut microbiome, we further addressed our hypothesis, whether probiotics can maintain gut microbiome homeostasis in members during a long stressful voyage. The structures of the gut microbiota of subjects in the  PB group were compared (Bray–Curtis distances) based on metagenomic genus and species level, Fig. 1A, D, but we did not observe any significant differences. To confirm our observation, we further compared the microbial structure of sailors between PCB group and PB group at T1 and T2 time points. Interestingly, no significant difference was found at baseline between PCB_T1 and PB_T1, but the compositions of the gut microbiota of the two groups were highly distinct at the end of the ship voyage T2, which confirmed the positive impact of the probiotics consumed. Even though the change in microbial structure was limited, we could also observe some specific changes at the microbial genus and species level. The css + log abundances of the genus *Alteromonas, Prevotella* and *Sinorhizobium* decreased significantly (Fig. 2A). Whereas species *Clostridium* bacterium 1_7_47FAA and *Clostridium bolteae* increased significantly in the probiotic group (Fig. 3A) at the end of ship voyage T2 (Wilcoxon rank-sum tests).

### Alteration in metabolism during the ship voyage

High-quality reads from all samples were assembled and annotated for protein-coding genes by MOCAT2 and STAMP for functional profiles, indicating the number of sequences allocated to various pathways in order to investigate the observed variations in functional profiles of gut microbiota during the long ship voyage.

**Figure 4:**
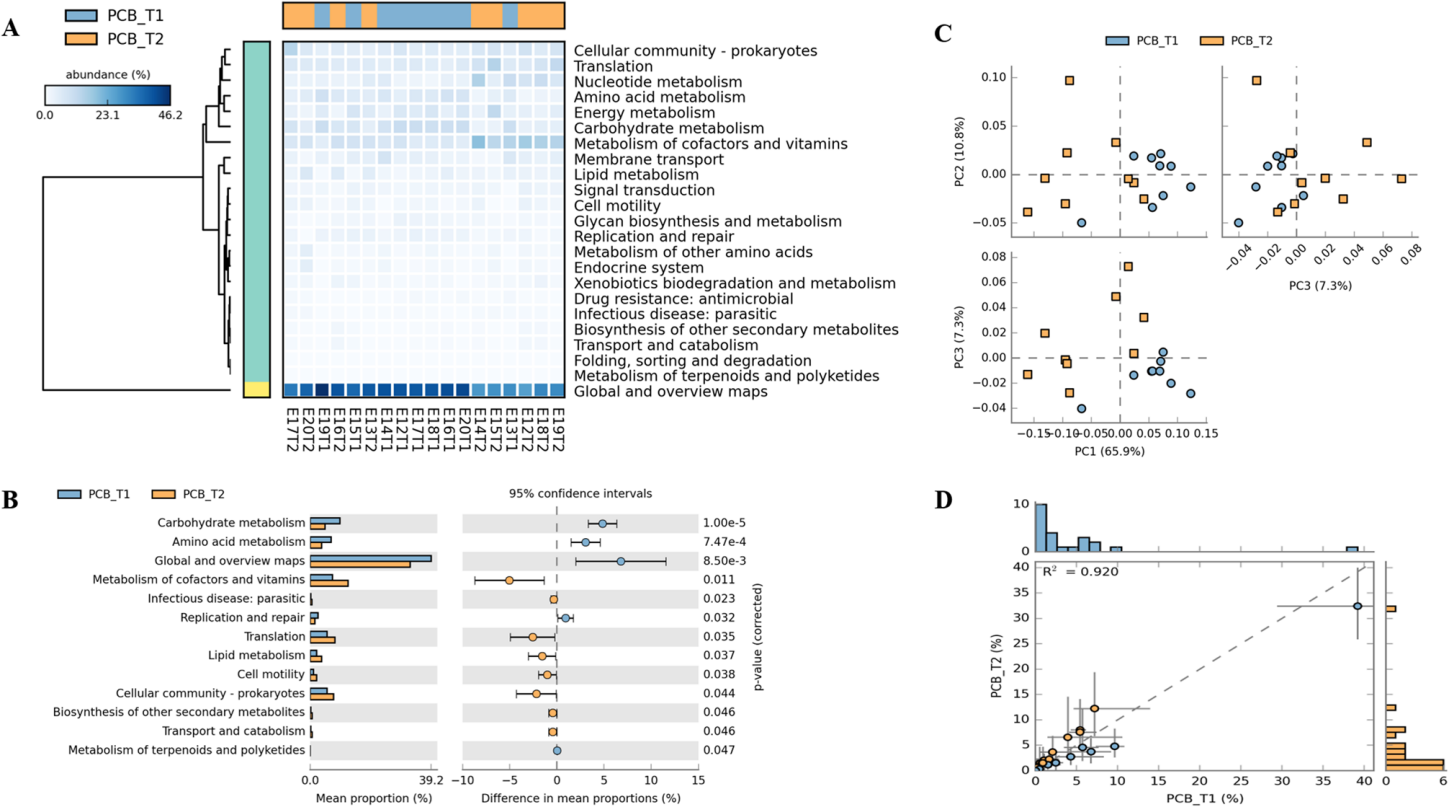
Metagenomic profile comparisons of metabolic pathways associated with PCB group members determined using STAMP analysis (Level-2). **A** - Expression of all altered pathways. **B** - Positive differences between proportions denote greater abundances in the PCB_T1 timepoint (blue), whereas negative differences between proportions show greater abundances in the PCB_T2 timepoint (orange) for the given pathways. Corrected *p*-values were calculated based on two-sided Welch’s t-test no correction and Welch’s inverted CI method. Difference with a *p*-value of < 0.05 were considered to be significant. **C** - PCA plot determined from the proportion of reads assigned to each pathway within a sample. **D** - Profile scatter plot with histograms showing the proportion of genes and functional profile, relative to all assigned genes, within the PCB group at both the time points.

We predicted functional profile at levels 1, 2 and 3 in both PCB (Supplementary Table S11) and PB groups (Supplementary Table S12). At level -2 in PCB group (Fig. 4) heatmap and differentially abundant (DA) pathway indicated the proportion of sequences assigned to each pathway for example, carbohydrate metabolism, amino acid metabolism, metabolism of cofactors and vitamins, etc. (Fig. 4A, B). PCA was performed based on the two-sided Welch’s t-test of the gut microbial functional gene profiles in  PCB group (Fig. 4C). The specific changes in the microbial metabolic pathways were represented by an increase in lipid metabolism, vitamin and cofactor metabolism, and decreased carbohydrate and amino acid metabolism (Fig. 4B). The mean proportion of sequences within each group were assigned to each pathway, more pathways were enriched in PCB_T2 group as compared to baseline PCB_T1 (Fig. 4D).

### Probiotic maintained metabolism during long ship voyage

**Figure 5:**
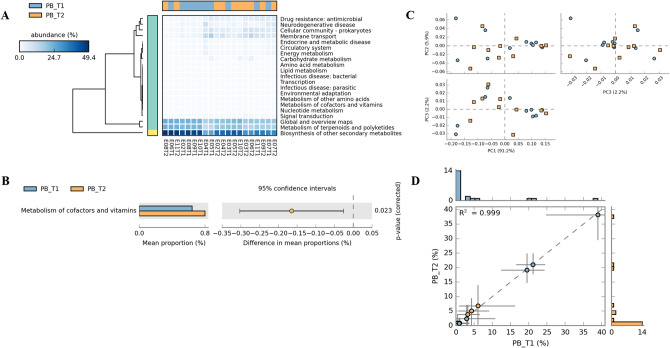
Metagenomic profile comparisons of metabolic pathways associated with PB group members determined using STAMP analysis (Level-2). **A** - Expression of all altered pathways. **B** - Positive differences between proportions denote greater abundances in the PB_T1 timepoint (blue), whereas negative differences between proportions show greater abundances in the PB_T2 timepoint (orange) for the given pathways. Corrected *p*-values were calculated based on two-sided Welch’s t-test no correction and Welch’s inverted CI method. Difference with a *p*-value of < 0.05 were considered to be significant. **C** - PCA plot determined from the proportion of reads assigned to each pathway within a sample.  **D** - Profile scatter plot with histograms showing the proportion of genes and functional profile, relative to all assigned genes, within the PB group at both the time points.

We observed that the stressful ship voyage has a major effect on the gut microbiome as indicated in the PCB group. We were interested to know whether probiotics intervention would maintain homeostasis of the gut microbiota in expedition members during the journey. No major significant alteration was observed in PB group gut microbiota composition during ship voyage as mentioned in the above section. However, there was a difference in gut microbial structure of   PCB and the PB group members at baseline as they were healthy with distinct microbial compositions. Further comparison of PB group between T1 and T2 did not show any significant alterations (Fig. 5C). However, the intestinal microbiota structure of two groups (PCB_T2 and PB_T2) was highly distinct at the end of the sea voyage, confirming the positive effects of the probiotics. The alterations in the gut metabolic pathways in PB group at the end of ship voyage was limited and non-significant, metabolic homeostasis was maintained with minor alterations observed in the metabolism of terpenoids and polyketides, biosynthesis of other secondary metabolites, metabolism of cofactors and vitamins at the end of ship voyage, etc. (Fig. 5A, B). Same number of enriched pathways  were observed in PB_T1 and PB_T2 (Fig. 5D).

### Alteration of gut microbial carbohydrate active enzymes (CAZymes)

**Figure 6:**
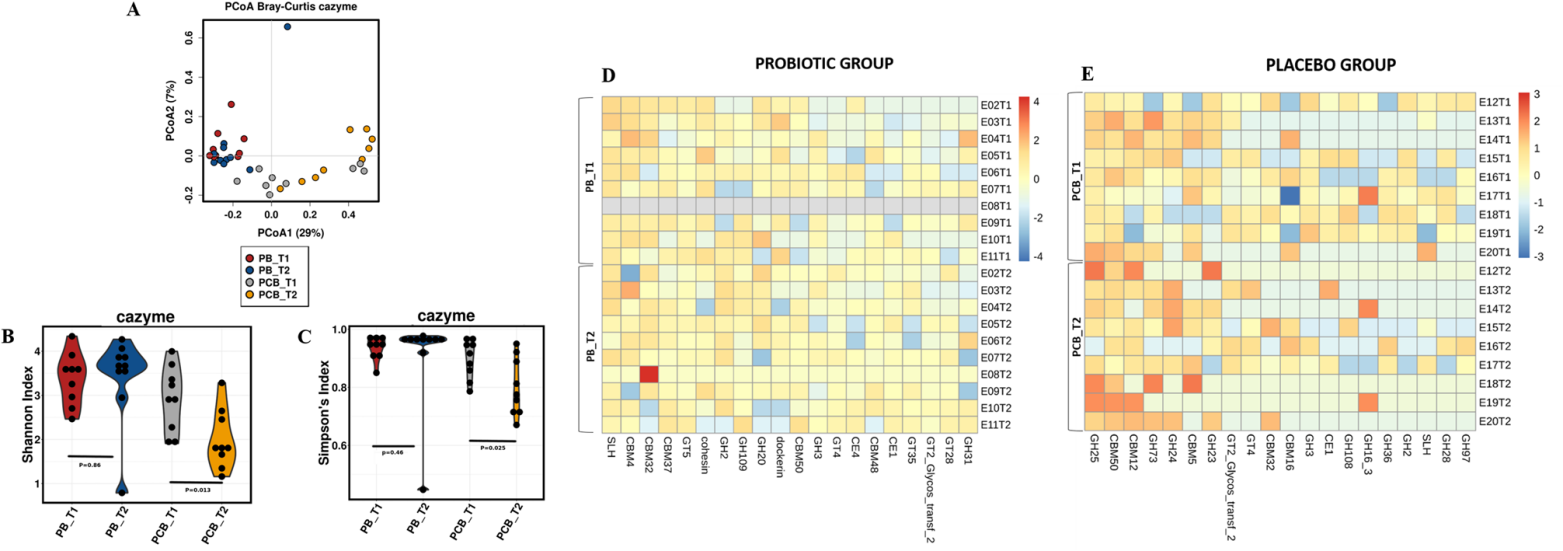
Alterations of the gut microbial CAZy genes during the stressful ship voyage. **A** - The Bray–Curtis distance-based gut microbial CAZy gene profile of members during the ship voyage. The points in different colors represent the intestinal microbial structure of the subjects in each group. **B**, **C** - The alpha diversity, including Shannon and Simpson indexes, of the gut microbial CAZy genes of expedition members both in PCB and PB groups at baseline and at the end of the ship voyage. Significant change was observed between PCB_T1 and PCB_T2 (*p* < 0.05).  **D**, **E** - The heatmap showing css + log transcformed abundance of top 20 abundant CAZy gene families, including glycoside hydrolases (GH) and carbohydrate binding moiety (CBM) in PCB group and PB group during the ship voyage. CAZy genes were filtered at E-value < 1e–18 and coverage > 0.35 for all bacteria using dbCAN HMMdb.

A shift in the gut microbial CAZy gene profile (Supplementary Table S13) based on the Bray–Curtis distances (Fig. 6A) and a significant decline in the alpha diversity of microbial CAZy genes (Fig. 6B, C) were observed at the end of the voyage in PCB group, which were represented by a decrease in the relative abundance of the gene families glycoside hydrolases (GH), glycosyltransferases (GT) and carbohydrate binding module (CBM) (Fig. 6E). These results showed that the stressful ship voyage disrupted the homeostasis and functional characteristics of the gut microbiota.

### Probiotics stabilized the gut microbial carbohydrate active enzymes (CAZymes)

There was a limited and non-significant alteration in the gut microbial metabolic pathways in the PB group during the ship voyage (Supplementary Table S12). No significant change was observed in the diversity and abundance of microbial CAZy genes (Fig. 6B–D). Taken together, these findings indicate that during the stressful ship voyage, the probiotics maintained the gut microbial homeostasis.

### Correlations and linear regresssion analysis between the microbial taxa and sea sickness

**Figure 7:**
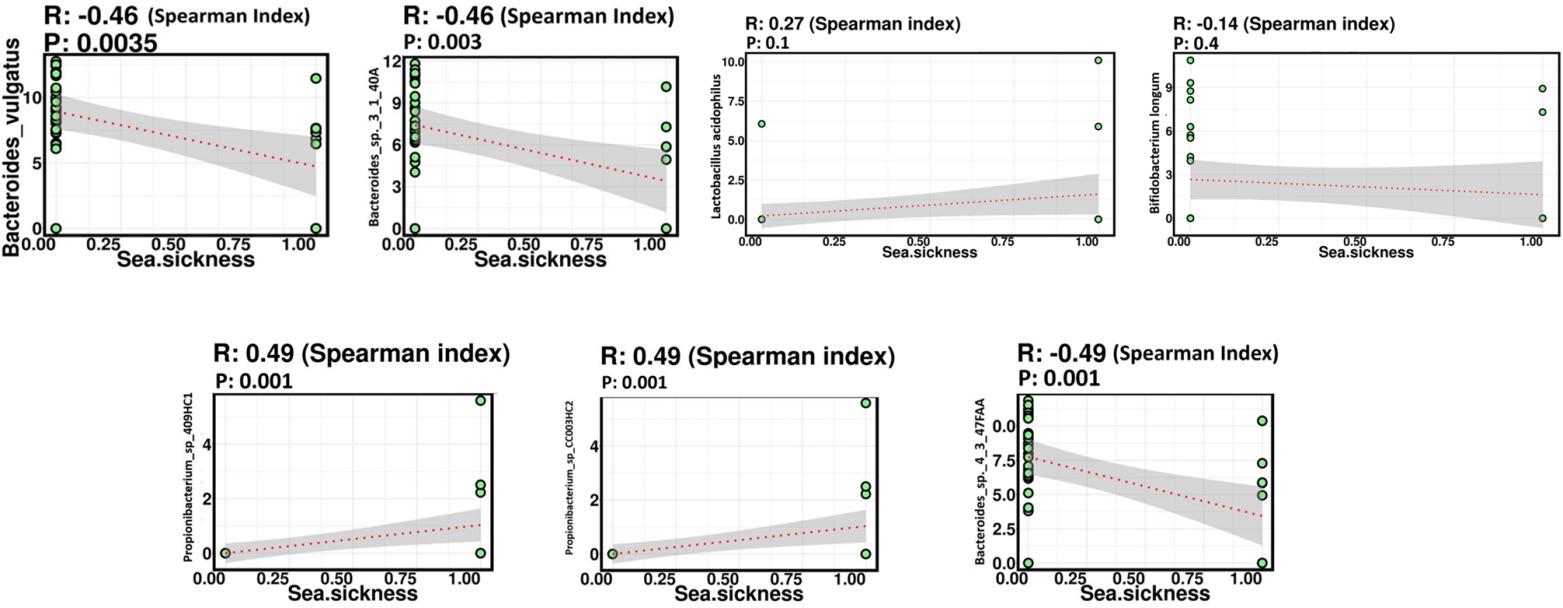
Spearman correlations indicate significant (positive and negative) relationships between microbial species and sea sickness. *r: Spearman's correlation coefficient*. Species of genus *Propionibacterium* (r = 0.49, *p* = 0.001) show strong positive correlation while that of genus *Bacteroides* (r = − 0.49 and − 0.46, *p* = 0.001 and 0.003) show negative correlation with sea-sickness. *Lactobacillus acidophilus* was positively correlated (r = 0.27, *p* = 0.1) and *Bifidobacterium longum* was negatively correlated (r = − 0.14, *p* = 0.4). *p*-value < 0.05 was considered statistically significant.

We explored the correlation between microbial taxa at various levels and sea sickness. The correlation analysis  was determined by using  the Spearman correlation coefficient. Comparing the relative abundances, both positive and negative correlations existed between gut microbial species and sea sickness (Fig. 7 and Supplementary Fig. S2). Of top 10 significant species, *Propionibacterium*_sp._409HC1 (*r* = 0.49, *p* = 0.002), *Propionibacterium* sp._CC003HC2 (*r* = 0.49, *p* = 0.002), and *Pseudomonas putida* (*r* = 0.39, *p* = 0.014) showed strong and significant positive correlation, whereas *Lachnospiraceae*_bacterium_1_1_57FAA, *Lachnospiraceae*_bacterium_8_1_57FAA, *Methanobrevibacter smithii* (*r* = − 0.4, *p* = 0.013), *Bacteroides vulgatus*, *Bacteroides*_sp._3_1_40A (*r* = − 0.46, *p* = 0.004), *Bacteroides*_sp._4_3_47FAA (*r* =− 0.49, *p* = 0.002), exhibited significant and strong negative correlation. Supplementary Tables S14 and S15 show taxa associated with sea sickness at genus and species level, respectively. Mixed effect linear regression analysis of the gut microbiota profiles and sea sickness after completion of 24 days ship voyage identified significant alterations in the abundance of microbiota. After completion of the ship voyage in sea sickness affected individuals of both the groups, *Bacteroides* (*p* = 0.0082), *Collinsella* (*p* = 1.9 e^−05^), *Parabacteroides* (*p* = 1.4 e^−05^) and *Subdoligranulum* (*p* = 4.4 e^−07^) followed a similar trend and had decreased abundance. On the other hand, *Clostridium* (*p* = 9.3 e^−06^), *Coprococcus* (*p* = 2.4 e^−07^), *Propionibacterium* (*p* = 0.027), *Pseudomonas* (*p* = 2.3 e^−05^), *Roseburia* (*p* = 2.3 e^−06^), *Ruminococcus* (*p* = 0.0001) had increased abundance as compared to non-affected individuals in both the groups (Supplementary Fig. SF3). The alterations observed in the abundance of various microbial genera was statistically significant. Similarly, we analyzed the mixed effect linear regression analysis of microbiota profiles with multiple phenotypic parameters such as headache, nausea, vomiting, smoking and diet. The detailed description of significant alterations in the genus after multiple testing using FDR and Bonferroni correction is represented in Supplementary Table S16.

## Discussion

The current study describes the gut metagenomic analysis of 19 participants from 38th ISEA on a ship voyage to Antarctica. The study was designed to explore the effects of stressful and extreme environmental conditions of Antarctica on the gut microbiota composition and diversity after the probiotic intervention. The subjects were randomly divided into PCB and PB groups and their fecal samples analyzed for microbial sequencing. During ship voyages in extreme environmental conditions, sea sickness is the most prominent physiological condition experienced by expedition members^4,33–35^. Sea sickness is associated with nausea, vomiting, headache, drowsiness, sleep deprivation and increased irritability which ultimately affects the individuals' physiological health and physical performance. These symptoms can be correlated with decreased dietary intake, environmental conditions and regular vomiting leading to quantitative and qualitative changes in gut microflora. The changes could be attributed to nutrients absorption or intestinal microbial metabolite secretions, resulting in disturbance and decline in normal flora and overgrowth of potential pathogens^36,37^. In the present study, data suggest the occurrence of 44% sea sickness in PCB group as compared to 10% in PB group, which is far less than the previously reported sea sickness by the same research group on similar expeditions^4,34^. Thus, probiotic helped members to reduce sea sickness and maintain overall gut homeostasis.

It is believed that the human gut microbiome is predominantly determined by various factors such as host genetics, environmental microbial flora, daily diet and antibiotics^38–40^. But during the ship voyage to Antarctica, members also experienced other factors like extreme environmental conditions such as high salinity (35% on an average), high humidity (90% to 100% on an average), intense UV radiation, storms, rolling and pitching of ship, monotonous environment, altered circadian rhythms, sleep deprivation^1,3,41^ etc., in addition to altered diet. The host appetite and food intake are correlated with the structure and function of the gut microbiome^42,43^. Since diet is considered as one of the key determinants of the composition and function of the gut microbiome^44^, along with other environmental factors, changed diet and unavailability of fresh fruits and vegetables during ship voyage, could have a profound effect on the structure and functionality of the individuals gut microbiome. The present study reveals substantial microbial alteration in PCB group individuals on stressful ship voyage which could be due to multiple factors contributing to the reduced diversity of gut microbiome. Though long term stays in space^45,46^, extreme environment and dietary alterations have been reported as major causative factors for dysbiosis in oral^4,21^ and gut microbiota^9^ within months, but there are no genotypic changes reported.

To restore dysbiosis in gut microbiota, probiotics could be a potential intervention. Therefore, in the current study, we demonstrated a probiotic cocktail as a dietary intervention, a better preparation than a single strain probiotic to restore and maintain the characteristics of native gut microbiota^47,48^. The probiotic cocktail contained a combination of four bacterial and one yeast species, including *Lactobacillus acidophilus*, *Lactobacillus rhamnosus*, *Bifidobacterium longum*, *Bacillus coagulans and Saccharomyces boulardii*. *Saccharomyces boulardii* (a yeast), after exposure to simulated gastric juice containing pepsin and hydrochloric acid, remains viable^49^. Its neuraminidase activity has been shown to have possible therapeutic effects in the rat model of gastric ulcer^50,51^ and, works to restore the normal microflora and modulates the microbiome by colonization throughout the susceptibility phase^52^. *Bifidobacterium* inhibits harmful bacteria, improves the function of gastrointestinal barrier, initiates protective functions against pathogens and increases the proportion of beneficial bacteria in the gut microbiota^53^. *Lactobacillus* are known for the production of antimicrobial substances, metabolites and bacteriocins. *Lactobacillus* species also prevent pathogen colonization in the gut ecosystem by mechanism of competitive exclusion for nutrients and attachment sites^54^, protecting the gut mucosal barrier from disruption^55^. Moreover, *Lactobacillus acidophilus*, *Lactobacillus rhamnosus*, *Bifidobacterium longum* can survive in the acidic environment of gut for longer durations^56,57^ which are the major components of our probiotic cocktail.

In the present study, the abundance of *Bacteroidetes* (*Bacteroides* sp. 4_1_36) and *Proteobacteria* (*Pseudomonas stutzeri*), were significantly higher in PCB group. *Pseudomonas stutzeri* is associated with the metabolism of lactate and may contribute to lactate intake and digestion in intestinal tissues^58^. This can be related to carbohydrate metabolism as evident from our results, where a significant alteration is observed in carbohydrate metabolism genes. After the completion of ship voyage the abundance of *Bacteroidetes* was higher in PB group as compared to PCB group. *Bacteroides vulgatus* can utilize and ferment a variety of complex polysaccharides such as amylose and amylopectin to simpler forms of carbohydrates for energy harvesting^59^. Kang et al.^60^ reported that patients with Crohn’s disease have decreased abundance of *Bacteroides vulgatus* as compared to healthy controls. In the present study, the abundance of *Bacteroides uniformis* decreased in PCB group during the expedition and was significantly higher in PB group at the end of ship voyage. *Bacteroides uniformis* shares glycans with human gut bacteria as it has an immunomodulatory activity which helps maintain the gut homeostasis^61^. *β-*glucans are the dietary nutrients present in oats, barley and mushrooms, which are readily available during the ship voyage^62^, significantly influence the changes in gut microbiota and thus play an intermediate role in maintaining health.

Our findings revealed alteration of metabolic pathways related to energy metabolism following 24 days of ship voyage under extreme environment in PCB group. Diverse microbial communities in the gastrointestinal tract are already established facts as they ferment carbohydrates into short-chain fatty acids (SCFAs) and lactate as a way of producing ATP^63^. Higher energy to the host can be provided by better regulation of metabolites production by commensal and probiotic bacteria^64^. Functional analysis revealed that overall metabolism in PCB group was significantly altered, including metabolism of carbohydrates, amino acids, cofactors and vitamins, and lipids. It has been reported that carbohydrate metabolism can be altered due to stress^65^, whereas altered amino acid metabolism can induce inflammation^4^. On the basis of current data, it is tempting to speculate that, after 24 days of ship travel, PB supplementation may have optimized the metabolic potential of gut microbiota in PB group, maintaining the stability of taxonomic composition and functional potential. The results show that in PB group, the taxonomic abundance was almost similar to their respective baseline levels whereas, significant alterations were observed in PCB group. However, on the basis of the functional pathway analysis, PB group had beneficial metabolic adaptations compared to PCB group. This divergence in composition and functional profiling between PCB and PB group could be attributed to the beneficial effects of probiotic on the gut microbiome composition. The interesting part of the study was that the same samples were collected at two different time points, making it possible to compare microbial changes associated with the induction to different environments and easily compared with their baseline profile. Nguyen et al.^66^ recently reported that appropriate statistical methods accounting for the compositional nature of the data could help in avoiding false positivity. Thus, in the present study, the analysis was carried out using the latest pipelines to avoid false positivity, which provided more information for downstream data pooling and meta-analysis.

Taxonomic and functional analysis at genus and species level revealed significant changes in the gut microbiota composition, carbohydrate metabolism specifically, CAZy genes. Diversity of gut microbes and CAZy associated functional genes form the basis for maintaining homeostasis of the gut microbiome and host health^67^. The human gut microbiome encoded CAZy help in the degradation and digestion of fibers, complex carbohydrates and glycoconjugates to monosaccharides. The low diversity of CAZy associated gut microbes is closely linked to the proliferation of pathogenic and opportunistic pathogens, contributing to various chronic diseases. SCFA is produced by dietary polysaccharide, microbial fermentation and is associated with beneficial effects on the host^68^. Additionally, degrading complex carbohydrates to fermentable and energy-yielding SCFAs, the presence of large numbers of CAZy genes in the human gut enhances gut health directly or by cross-feeding mechanism^69^. *Bifidobacterium* and *Bacteroides* constitute approximately half of the CAZymes marker proportion. The above findings indicate that while the *Firmicutes* phyla (as a whole) have a higher prevalence of such efficient enzymes for energy harvesting, the existence of unique genera belonging to the *Bacteroidetes* and *Actinobacteria* phyla may also increase the energy-harvesting capabilities of the gut, which is evident from this study indicating the abundance of *Firmicutes* (*Clostridium* bacterium 1_7_47FAA and *Clostridium bolteae*) in PB group. It has been reported that *Clostridium bolteae* is associated with lean individuals^70,71^, which is positively correlated with our findings, as after 24 days of ship voyage, the average weight of PB group was decreased, which was found to be increased in PCB group with respect to their baseline weight. Similarly, compared to baseline data, individuals in PB group had decreased BMI, which may be the potential explanation for significant abundance of *Clostridium bolteae* in PB group. *Bacteroides* and *Clostridia* are known as important regulators of gut microbiome homeostasis^72^.

Recent evidence indicates that SCFAs modulate host metabolic health through a variety of tissue-specific pathways related to appetite regulation, energy intake, glucose homeostasis and immunomodulation. By controlling the luminal pH, mucus development, fuel for epithelial cells, and effects on immune function, microbial SCFAs production is necessary for gut barrier integrity^73^. They also promote mucin, defensin, and antibacterial peptide secretion, thereby regulating epithelial barrier function and bacterial endotoxin translocation^74^. The probiotic thus supports the growth of beneficial microbes and inhibits the proliferation of pathogenic and opportunistic pathogens, which, by stimulating natural immunity, contribute to the balance of microbiota, which is necessary for maintaining host health^75^.

In conclusion, stressful ship voyage is a major cause for sea-sickness that altered the gut microbiota composition and diversity of its functional characteristics in expedition members. Use of probiotics during the ship voyage regulated the gut microbiota homeostasis and reduced the prevalence of sea sickness and other physiological complications. To the best of our knowledge this is the first evidential study using probiotics intervention for Antarctic expedition members. The results of this study will add to our knowledge and provide a feasible solution for maintaining gut health during ship voyage to Antarctica and provide new insights into the use of probiotics as a nutritional intervention.

### Limitations and merits

The study’s design and subsequent data interpretation are limited due to the small subject size, limiting the statistical power. But because of logistic issues, the availability of a small subject size is unavoidable. However, the study's merit is that it is the first of its kind, presenting a unique and novel exploration of the role of probiotics in maintaining gut microbiome homeostasis during a stressful ship voyage to Antarctica.

## Conclusion

The present longitudinal study involves 19 healthy male Indian members who participated in the 38th Indian Scientific Expedition to Antarctica via ship voyage. Stressful ship voyage is a major cause for sea-sickness that altered the gut microbiota composition and diversity of its functional characteristics in expedition members. This study is the first of its kind, where sea-sickness has been taken into consideration. Previous expedition studies reported sea-sickness causing severe discomfort to the participants affecting their work performance, psychological behavior, sleep pattern, etc. This kind of discomfort leads to refusal by participants to travel by ship and preferred to take air journey, causing logistic problems to the authorities. Therefore, the probiotic intervention was proposed for better management during the ship journey. Fortunately, probiotics maintained intestinal microbiome homeostasis and further prevented sea sickness during the sea voyage. The use of probiotics during the ship voyage regulated the gut microbiota homeostasis and reduced the prevalence of sea sickness and other physiological complications. The results of this study will add to our knowledge and provide a feasible solution for maintaining gut health during ship voyage to Antarctica and provide new insights into the use of probiotics as nutritional intervention.

## Supplementary Information

Supplementary Information.

Supplementary Legends.

Supplementary Figure S1.

Supplementary Figure S2.

Supplementary Figure S3.

## Data Availability

The sequence data from this study are deposited in the GenBank Sequence Read Archive with the accession number PRJNA694018.
