# Supplementary material for: Probiotics maintain the gut microbiome homeostasis during Indian Antarctic expedition by ship
Source: Sci Rep. 2021 Sep 22;11:18793. doi: 10.1038/s41598-021-97890-4 (PMC8458292; doi:10.1038/s41598-021-97890-4)
Supplement: Supplementary file 2 — Supplementary Legends. [file 41598_2021_97890_MOESM2_ESM.docx]

**Supplementary figure legends**

**Supplementary Figure SF1:**

Relative abundance of intestinal microbes of each individual at A) phylum and B) genus level in both the placebo (PCB_T1 and PCB_T2) and probiotic (PB_T1 and PB_T2) group at baseline and after the completion of ship voyage.

**Supplementary Figure SF2:**

Spearman correlation (positive and negative) of gut microbial species and sea-sickness.

**Supplementary Figure SF3:**

Mixed effect linear regression analysis of the gut microbiota profiles (at genus level) and sea-sickness after completion of 24 days of ship voyage (T2).
