# Supplementary figures and images for: Probiotics maintain the gut microbiome homeostasis during Indian Antarctic expedition by ship
Source: Sci Rep. 2021 Sep 22;11:18793. doi: 10.1038/s41598-021-97890-4 (PMC8458292; doi:10.1038/s41598-021-97890-4)

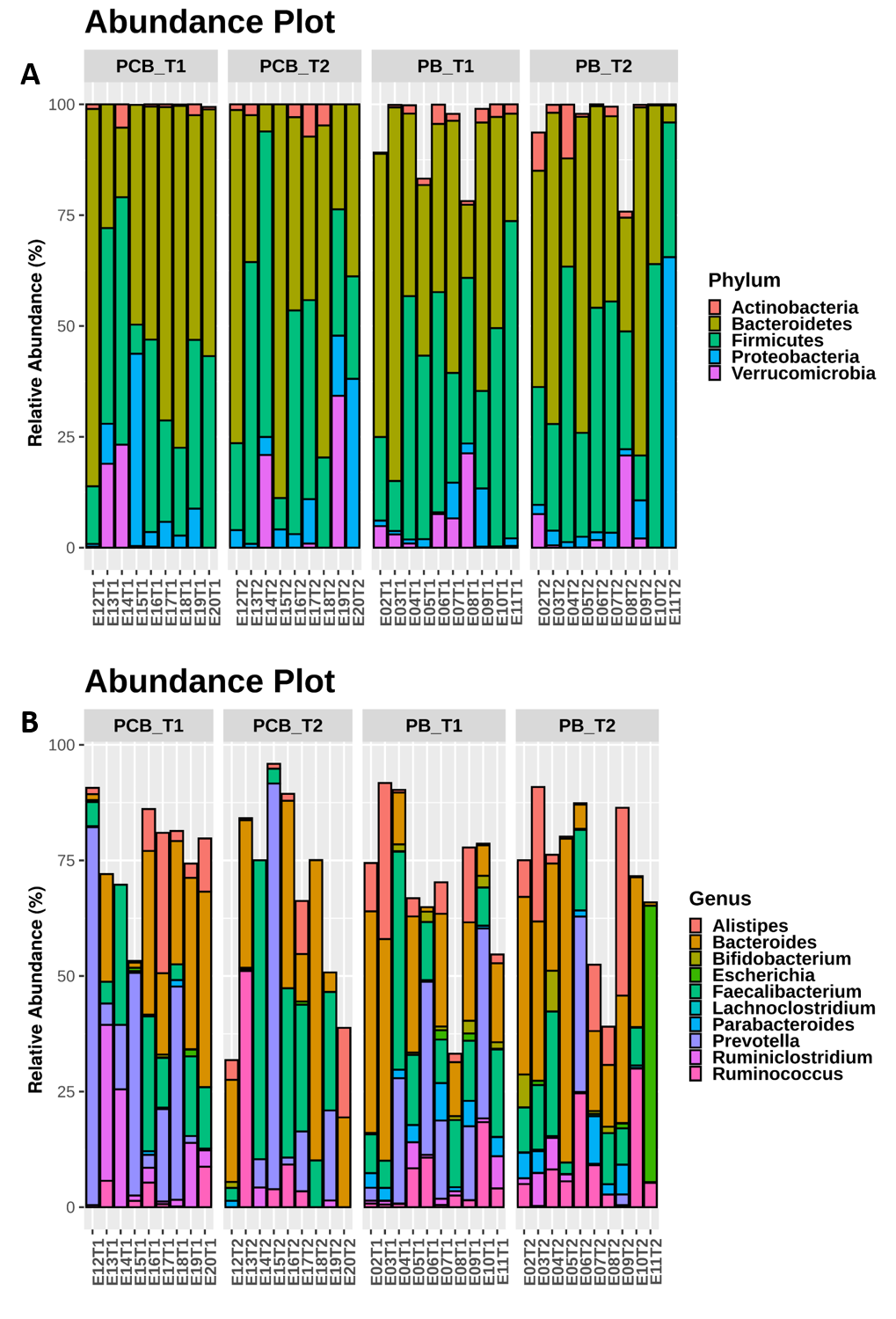

Supplement: Supplementary file 3 — Supplementary Figure S1. [file 41598_2021_97890_MOESM3_ESM.tif]

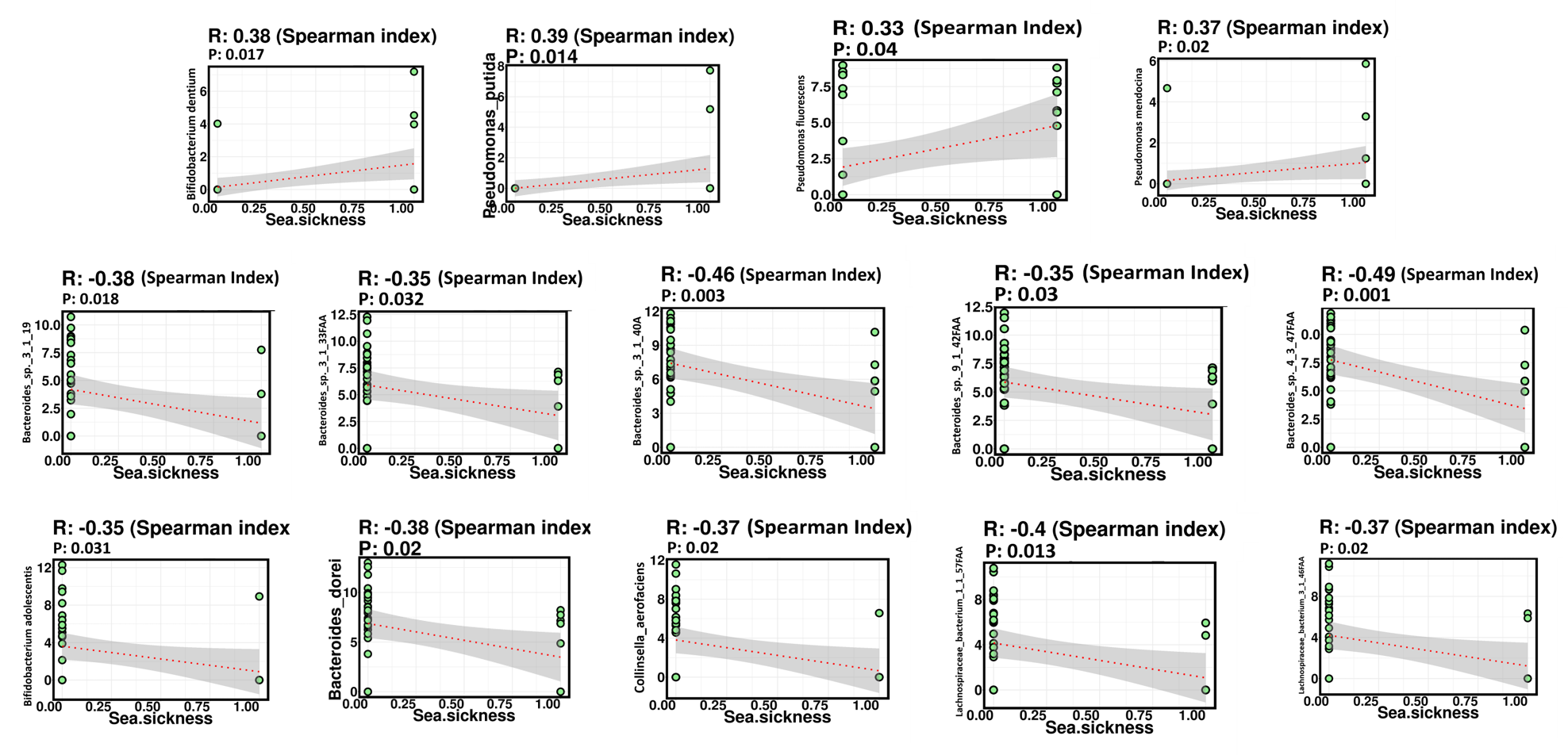

Supplement: Supplementary file 4 — Supplementary Figure S2. [file 41598_2021_97890_MOESM4_ESM.tif]

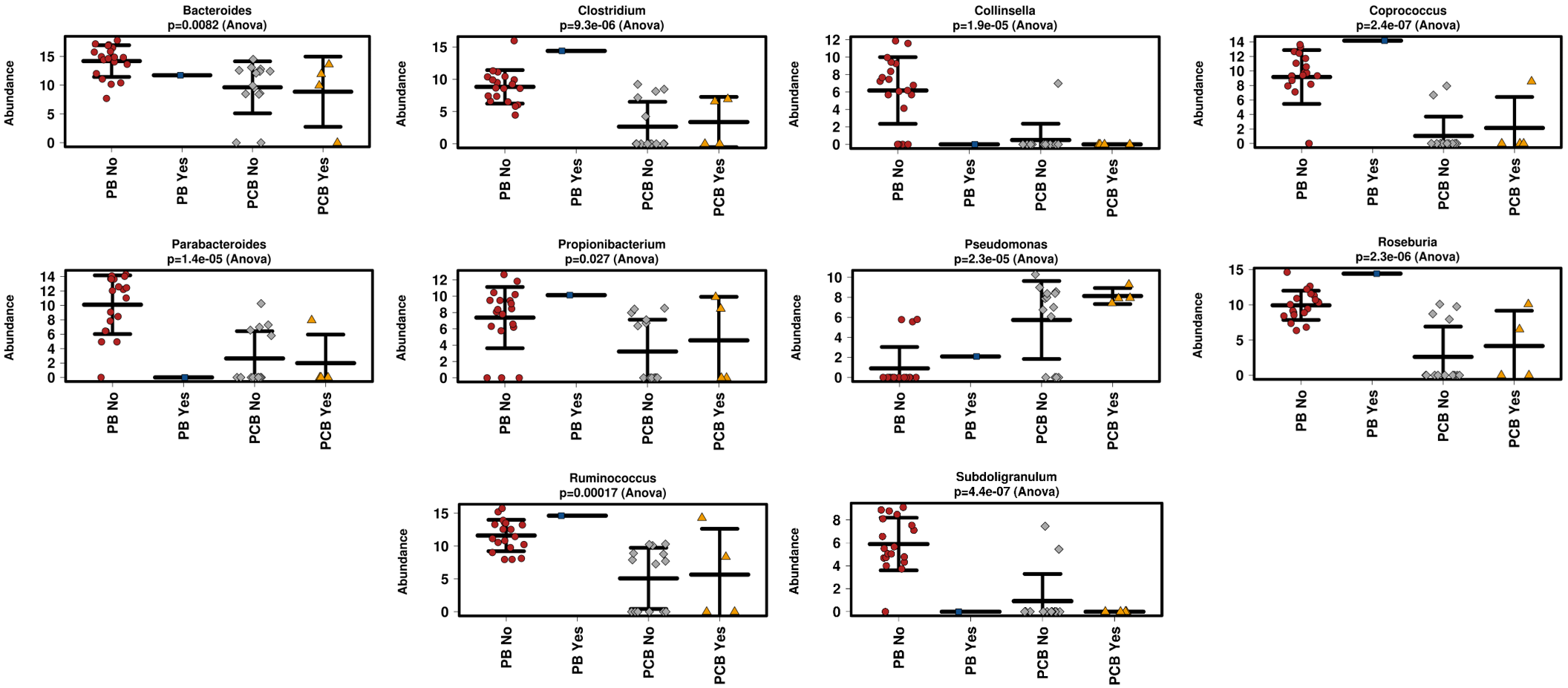

Supplement: Supplementary file 5 — Supplementary Figure S3. [file 41598_2021_97890_MOESM5_ESM.tif]
